# A phase I study of the nitroimidazole hypoxia marker SR4554 using ^19^F magnetic resonance spectroscopy

**DOI:** 10.1038/sj.bjc.6605425

**Published:** 2009-11-24

**Authors:** C P Lee, G S Payne, A Oregioni, R Ruddle, S Tan, F I Raynaud, D Eaton, M J Campbell, K Cross, G Halbert, M Tracy, J McNamara, B Seddon, M O Leach, P Workman, I Judson

**Affiliations:** 1 Cancer Research UK Centre for Cancer Therapeutics, Cancer Research UK Clinical Magnetic Resonance Research Group and Section of Medicine, The Institute of Cancer Research and Drug Development Unit, The Royal Marsden Hospital, 15 Cotswold Road, Sutton, Surrey SM2 5NG, UK; 2 Drug Development Office, Cancer Research UK, 61 Lincoln's Inn Fields, London WC2A 3PX, UK; 3 Cancer Research UK Formulation Unit, Strathclyde Institute of Pharmacy and Biomedical Sciences, Royal College Building, University of Strathclyde, 204 George Street, Glasgow G1 1XW, UK; 4 SRI International, 333 Ravenswood Avenue, Menlo Park, California 94025, USA

**Keywords:** tumour hypoxia, nitroimidazole, magnetic resonance spectroscopy, non-invasive

## Abstract

**Background:**

SR4554 is a fluorine-containing 2-nitroimidazole, designed as a hypoxia marker detectable with ^19^F magnetic resonance spectroscopy (MRS). In an initial phase I study of SR4554, nausea/vomiting was found to be dose-limiting, and 1400 mg m^−2^ was established as MTD. Preliminary MRS studies demonstrated some evidence of ^19^F retention in tumour. In this study we investigated higher doses of SR4554 and intratumoral localisation of the ^19^F MRS signal.

**Methods:**

Patients had tumours ⩾3 cm in diameter and ⩽4 cm deep. Measurements were performed using ^1^H/^19^F surface coils and localised ^19^F MRS acquisition. SR4554 was administered at 1400 mg m^−2^, with subsequent increase to 2600 mg m^−2^ using prophylactic metoclopramide. Spectra were obtained immediately post infusion (MRS no. 1), at 16 h (MRS no. 2) and 20 h (MRS no. 3), based on the SR4554 half-life of 3.5 h determined from a previous study. ^19^Fluorine retention index (%) was defined as (MRS no. 2/MRS no. 1)*100.

**Results:**

A total of 26 patients enrolled at: 1400 (*n*=16), 1800 (*n*=1), 2200 (*n*=1) and 2600 mg m^−2^ (*n*=8). SR4554 was well tolerated and toxicities were all ⩽grade 1; mean plasma elimination half-life was 3.7±0.9 h. SR4554 signal was seen on both unlocalised and localised MRS no. 1 in all patients. Localised ^19^F signals were detected at MRS no. 2 in 5 out of 9 patients and 4 out of 5 patients at MRS no. 3. The mean retention index in tumour was 13.6 (range 0.6–43.7) compared with 4.1 (range 0.6–7.3) for plasma samples taken at the same times (*P*=0.001) suggesting ^19^F retention in tumour and, therefore, the presence of hypoxia.

**Conclusion:**

We have demonstrated the feasibility of using ^19^F MRS with SR4554 as a potential method of detecting hypoxia. Certain patients showed evidence of ^19^F retention in tumour, supporting further development of this technique for detection of tumour hypoxia.

Nitroimidazoles were initially developed as radiosensitising agents and later as cytotoxins for hypoxic cells. There is also interest in their use as hypoxia markers (Chapman, 1991; Hodgkiss, 1998). This is based on the ability of nitroimidazoles to undergo selective bioreductive metabolism by nitroreductases under hypoxic conditions to reactive intermediates, which then become covalently bound to macromolecules within the cell (Wardman, 1977; Varghese and Whitmore, 1980). These adducts are retained within the hypoxic cells for longer periods of time as compared with the parent compound (Chapman *et al*, 1981; Miller *et al*, 1982), which forms the basis for detection of bioreduced nitroimidazoles in hypoxic cells. The side chain of the imidazole ring also becomes bound in hypoxic cells (Raleigh *et al*, 1985); thus labelling of the side chain with an appropriate isotope or immunologically recognisable marker allows the bioreduced compound to be detected, indicating the presence of hypoxia.

SR4554 is a 2-nitroimidazole that has been rationally designed for use as a non-invasive probe of tumour hypoxia (Aboagye *et al*, 1995b, 1997a, 1997b, 1998a, 1998b). The key properties introduced to produce the desired chemical and pharmacokinetic properties for a hypoxia marker are (1) a nitro group with the appropriate reduction potential for hypoxia-dependent nitroreduction; (2) amide and hydroxyl substituents in the side chain designed to increase hydrophilicity, resulting in increased water solubility, rapid renal excretion and reduced central nervous penetration and hence toxicity; and (3) three magnetically equivalent fluorine atoms to increase the sensitivity of detection by ^19^F magnetic resonance spectroscopy (MRS).

Extensive preclinical studies have validated the use of SR4554 as a hypoxia marker. In preclinical tumour models, for which plasma half-life was 37 min (Seddon *et al*, 2002), the 3-h ^19^F retention index (^19^F-RI), defined as the ratio of total SR4554 tissue concentration (determined by MRS) at 3 h divided by that at an early time point (0.75–1 h) after administration of SR4554, expressed as a percentage, was found to correlate well with pO_2_ values measured by the polarographic electrode (Seddon *et al*, 2002). Thus, the 3-h ^19^F-RI was thought to be a good measure of retained SR4554 bioreduction products and therefore of hypoxia in these models. The ^19^F-RI was also shown to correlate with the intrinsic radiobiological hypoxic fraction of several mouse tumours (Seddon *et al*, 2002). Furthermore, in experiments using oxygen-modifying agents such as hydralazine, combretastatin or carbogen+nicotinamide, the change in the 3-h ^19^F-RI was shown to correlate very closely with changes in pO_2_ values determined using Eppendorf probes (Seddon *et al*, 2002).

These promising preclinical data led to the commencement of a phase I safety study of SR4554 in patients with advanced malignancies, in 2000, under the auspices of Cancer Research UK. This study enrolled eight patients and reported nausea and vomiting as dose-limiting toxicities (DLTs) at a dose of 1600 mg m^−2^ (Seddon *et al*, 2003). No other toxicities were observed. Pharmacokinetic studies demonstrated a short plasma half-life (3.45±0.62 h), rapid plasma clearance (12.6±3.4 l h^−1^) and high renal excretion (87.4±8.6%), the ideal profile for a hypoxia marker (Seddon *et al*, 2003). Preliminary MRS studies of three of these patients showed promising results, with some evidence of ^19^F retention.

This study was therefore continued, and we now report further data obtained from this phase I study of SR4554 using ^19^F MRS.

The primary aim of this part of the study was to further investigate ^19^F MRS to develop a reliable parameter of SR4554 retention in tumour that could be used as a potential surrogate measure of tumour hypoxia. This involved the correlation of tumour ^19^F signal with plasma SR4554 levels and of the necessity achieving a dose of SR4554 capable of producing a ^19^F signal of sufficient intensity to be reliably detected by localised MRS. Variable success at the dose defined by the initial safety study led to a limited further dose escalation of SR4554 made possible by administration of a prophylactic antiemetic. Both unlocalised and localised spectroscopy were investigated.

## Materials and methods

### Study design

This was a single-centre, phase I study conducted under the auspices of Cancer Research UK. The study protocol was approved by the Cancer Research UK Internal Review Board and by the Royal Marsden Hospital Ethics Committee and Committee for Clinical Research.

All patients had a baseline dynamic contrast-enhanced MRI scan within 7 days of SR4554 infusion to provide information on tumour location and size. Patients received an intravenous (IV) infusion of SR4554 on day 1 followed by a total of 2–3 MRS studies over the subsequent 24 h. The first MRS study (MRS no. 1) was performed immediately after the SR4554 infusion; the second (MRS no. 2) at approximately 16 h post infusion and a third (MRS no. 3) at approximately 20 h post infusion if indicated.

Plasma and urine samples were collected over 24 h following SR4554 infusion for pharmacokinetic studies.

### Patient eligibility

Inclusion criteria for this study were (1) histologically proven solid malignancy for which patients were not receiving any anticancer treatment at the time of study other than cytokine or hormonal treatments; (2) tumours at least 3 cm in size at a site suitable for ^19^F MRS, that is, at no more than 4-cm depth; (3) age ⩾18 years; (4) life expectancy of at least 3 months; (5) WHO performance status ⩽2; (6) no change in corticosteroid requirement during the 2 weeks prior to study entry; (7) adequate haematological and biochemical function, that is, haemoglobin ⩾9 g dl^−1^, white cell count >3.5 × 10^9^ l^−1^, neutrophil >1.5 × 10^9^ l^−1^, platelets ⩾100 × 10^9^ l^−1^, plasma creatinine <130 *μ*mol 1^−1^, bilirubin <30 *μ*mol l^−1^, ALT and AST less than 2.5 × the upper limit of the normal range (ULN) if no known metastatic liver disease, or less than 5 × the ULN for patients with liver metastases; (8) written informed consent; and (9) agreement to the number of MRS scans required and ability to comply with this.

Exclusion criteria were (1) treatment with metronidazole at study entry; (2) radiotherapy during the previous 4 weeks or chemotherapy during the previous 3 weeks prior to study entry; (3) pregnant or lactating women; (4) female patients of child-bearing potential were required to have a negative serum pregnancy test prior to trial entry, and must have agreed to using medically approved contraceptive measures during the study and for 6 months subsequently; male patients must have agreed to using barrier contraception; (5) major thoracic and/or abdominal surgery during the previous 3–4 weeks from which the patient had not yet recovered; (6) patients with implanted or prosthetic magnetic materials, cardiac pacemakers or other equipment or aids, which were likely to be disturbed by the magnetic or radiofrequency fields of the magnetic resonance scanner; (7) patients with a known history of anaphylactic or anaphylactoid reaction to any contrast agent, including iodinated agents and gadolinium complexes; and (8) patients who were at poor medical risks because of concurrent non-malignant disease.

### SR4554 dosage and dose escalation

The initial safety study recommended 1400 mg m^−2^ as the maximum tolerated dose (MTD) (Seddon *et al*, 2003); hence in this study SR4554 was commenced at this dose level. In order to generate more reliable localised ^19^F MRS data, it was decided that higher doses of SR4554 should be explored following administration of prophylactic antiemetics in order to maximise ^19^F detection by MRS. Three higher doses of SR4554 were studied, 1800, 2200 and 2600 mg m^−2^. One patient was enrolled into each dose level provided there was no ⩾grade 2 toxicity seen, to a maximum dose of 2600 mg m^−2^. If DLT was observed, up to five further patients would be entered at the dose level below that at which DLT was seen, to allow evaluation and optimisation of the MRS scanning protocol.

Owing to the solubility of SR4554, which limited the maximum dose that could be administered in 1 l of 0.9% saline to 5.4 g, it was decided that 2600 mg m^−2^ would be the maximum dose to be explored, that is, close to the maximal deliverable dose in a 1 l volume for a patient with a body surface area of 2.0 m^2^. It was considered impractical to administer a diagnostic agent in a volume in excess of 1 l.

In the dose escalation part of the study, patients received prophylactic antiemetic with a single dose of metoclopramide (10 mg, p.o.) 0.5–1 h prior to SR4554 infusion. If the patient developed nausea or vomiting despite metoclopramide administration, granisetron (1 mg, p.o.) could be used to treat nausea in the same patient. For subsequent patients, granisetron (1 mg, p.o., 0.5–1 h before SR4554 infusion) could be used as prophylaxis.

If the maximum dose of 2600 mg m^−2^ was reached without any patient experiencing DLT, up to 10 patients could then be entered at this dose level to permit acquisition of localised MRS data.

SR4554 was administered intravenously either over 30 or 60 min (depending on body surface area, hence volume of infusion) as a single dose on day 1.

SR4554 was diluted with 0.9% normal saline to a total volume of 500 or 1000 ml. The drug was supplied by SRI International and formulated by Cancer Research UK Formulation Unit at the University of Strathclyde in 0.5 ml amber glass ampoules at a concentration of 200 mg ml^−1^ in 99% dimethyl sulphoxide (DMSO) and 1% Tween-80.

### Dose-limiting toxicity and MTD

Toxicities were graded using the National Cancer Institute Common Toxicity Criteria version 2.0.

Dose-limiting toxicity was defined as any of the following drug-related events: sensory or motor neuropathy>grade 1, neutropenia>grade 1, neutropenic fever, thrombocytopenia>grade 1, anaemia>grade 2, any non-haematological toxicity, including nausea and vomiting,>grade 1, and drug-related death.

Maximum tolerated dose was defined as the dose below that at which DLT occurred in any patient. Any neurological, haematological (with the exception of anaemia) or non-haematological toxicity greater than grade 1, was considered unacceptable for a diagnostic agent.

### Pre-treatment evaluation and follow up

Baseline evaluations (clinical assessment, haematological and biochemical laboratory tests) were performed within 1 week prior to patients receiving SR4554 and again on day 1. An electrocardiogram and a chest X-ray were performed within 4 weeks of starting the trial. Patients were then followed up with clinical assessment, haematological and biochemical laboratory tests weekly for 4 weeks. Patients who were to receive definitive treatment (chemotherapy, radiotherapy or surgery) for their disease were followed up for 2 weeks following SR4554 before being eligible to receive definitive treatment. Participation in this study was not allowed to delay a patient's definitive treatment. Patients who developed treatment-related toxicities had to be followed up until all toxicities had returned to baseline.

### Plasma pharmacokinetic study

Following SR4554 infusion, patients had plasma samples taken for analysis of SR4554 concentrations for up to 24 h on day 1 only. Samples were collected before treatment, during infusion, at the end of infusion, and then at 5, 10 and 60 min, and 4, 6, 16, 18, 20 and 24 h post infusion. Plasma samples were collected immediately before and after each MRS study to facilitate a comparison with the MRS-estimated concentrations of SR4554 in tumour to define the SR4554 ‘retention index’.

Blood samples (5 ml) were collected in heparinised tubes, centrifuged at 3000 r.p.m. (or 1200 **g**) at 4°C for 5 min and supernatants stored at −70°C until analysis. Plasma samples were extracted and analysed for presence of parent SR4554 by high-pressure liquid chromatography with UV detection (HPLC-UV), as previously reported (Aboagye *et al*, 1995a; Seddon *et al*, 2002). Pharmacokinetic data were analysed by non-compartmental analysis using WinNonLin software version 3.0 (Pharsight Corporation, Mountain View, CA, USA).

### Urinary excretion of SR4554

Urine was collected from each patient, over 24 h, immediately after the start of drug infusion, and total volume was measured and a 10-ml aliquot was stored at −70°C until analysis. SR4554 was extracted from urine samples and measured by HPLC-UV. Samples were prepared by spiking 250 *μ*l of urine with 30 *μ*l of internal standard (50 *μ*g ml^−1^) and adding 250 ml of 1% HCl and 1 ml of ethyl acetate. After centrifugation for 5 min at 5000 r.p.m., the organic phase was removed and dried, and the residue was dissolved in 150 *μ*l of methanol. Samples were then analysed for concentration of SR4554 as for plasma.

### Magnetic resonance spectroscopic studies

Magnetic resonance spectroscopic studies were performed using a 5, 10 or 16 cm dual-resonant ^1^H/^19^F transmit/receive surface coil and a 1.5 T MR system (Siemens Vision, Erlangen, Germany). Surface coils were built in the mechanical workshop at The Institute of Cancer Research. Transverse, sagittal and coronal localiser MR images were acquired using a TruFisp sequence (repetition time (TR)=6.3 ms; TE=3.0 ms). Magnetic resonance spectra localised only by the sensitivity profile of the RF surface coils (‘unlocalised’) were acquired in blocks of 512 scans using a 1.28 ms adiabatic radiofrequency pulse, and a repetition time (TR) of 1 s. The adiabatic pulse was used to ensure uniform signal excitation by the surface coil. Reference ^1^H spectra were acquired from tissue water using the same coil and sequence for use in subsequent SR4554 concentration calculations. Since water is present at high concentration, only eight scans were required for this. A longer TR (5 s) was used to remove the need to correct for saturation effects in the water spectra. If 512 scans of the unlocalised data did not show a clear ^19^F resonance peak, further unlocalised measurements were acquired to improve the signal-to-noise ratio (SNR); otherwise ^19^F spectroscopic images were acquired. Spectroscopic images used a grid size appropriate to the tumour, 8 × 8 × 8 phase-encoding steps, two scans per phase-encode, TR=1 s and the same adiabatic pulse as for the unlocalised measurements. It was not possible to also acquire ^1^H spectroscopic images, to be able to restrict the total examination time to an acceptable length. Spectral data were processed by apodisation, Fourier transformation, phase correction and peak integration. Peak integration was performed using the ‘Luise’ software supplied on the scanner (Siemens, Erlangen, Germany), which performs a best fit to the peak and baseline assuming a Lorentzian line shape.

Three parameters were calculated from the spectral data. The first was SNR (defined as peak height divided by peak-to-peak noise), used as an indicator of whether there was detectable ^19^F signal in the spectrum. Peaks were regarded as significant if SNR>1. The second parameter was the ^19^F-RI, defined as the ratio of the ^19^F signal measured at the second or third time point, relative to that measured at the first time point, where in each case the ^19^F signal was normalised by the signal from tissue water. This method has been previously validated in mouse model studies (Seddon *et al*, 2002). Finally we used the data from the unlocalised measurements to estimate the average SR4554 concentration in tissue (Payne *et al*, 2005). This is possible since the same surface coil was used for both ^19^F and ^1^H measurements, giving the same spatial sensitivity distribution for both measurements. At the second and third time points it is anticipated that SR4554 will only be retained in the regions of hypoxic tissues (i.e., part of the tumour) so that the average concentration over the sensitive region of the coil is much less than the actual concentration in the hypoxic regions. It is expected that SR4554 will be taken up into all tissues except fat, so only the water component of the ^1^H spectra was used in the calculation as follows: 
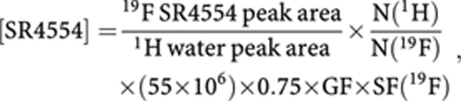
 where [SR4554] is the concentration of SR4554 (*μ*M), N(^1^H) is the number of ^1^H atoms in H_2_O (=2), N(^19^F) is the number of ^19^F atoms in SR4554 (=3), 55 × 10^6^ is the concentration of pure water (in *μ*M), 0.75 is the assumed water content of tissue and GF accounts for the difference in receiver gain used for the large water signal and small ^19^F signals. The final term, SF, is the ^19^F saturation factor and accounts for partial saturation of the signal expected from the shorter repetition time used in the ^19^F measurements, and is given by: 
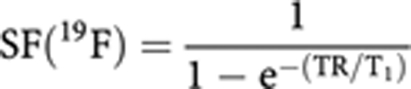


The T1 used was that measured at the same field strength in a pellet of liver microsomes containing SR4554.

The first MRS study (MRS no. 1) was performed immediately after infusion of SR4554. The second study (MRS no. 2) was performed 16 h post infusion. The choice of 16 h was based on the estimated time to virtually complete the elimination of SR4554 from plasma, that is, approximately five times the plasma elimination half-life of 3.5 h. At this time any significant signal would indicate retention of SR4554 bioreduction products indicative of hypoxia. In preclinical studies the optimal time point for calculating the ^19^F-RI was found to be 3 h, that is, approximately 5 × *T*_1/2_ in mouse (*T*_1/2_=37 min; Seddon *et al*, 2002). If a significant ^19^F signal was detected in MRS no. 2, a third MRS study (MRS no. 3) was performed between 18 and 20 h post infusion.

The position of the surface coil at the first MRS study for each patient was noted carefully to enable reproducible positioning for subsequent scans.

## Results

### Patient characteristics

A total of 26 patients with a variety of malignancies were enrolled between July 2003 and October 2005. Patient characteristics are shown in Table 1a. Most patients had gastrointestinal stromal tumour (GIST), head and neck cancer or melanoma. Other tumour types included sarcomas; fibromatosis; and cancers of the stomach, oesophagus and biliary tract. It was decided to include patients with a wide variety of tumour types in order to establish an MTD for SR4554 quickly, as there were recruitment difficulties in the study.

The median age of patients was 55 years (range 21–83). Four dose levels of SR4554 were investigated: 1400 (*n*=16), 1800 (*n*=1), 2200 and (*n*=1) 2600 mg m^−2^ (*n*=8).

Table 1b presents a summary of patients who experienced toxicity and the dose level of SR4554 they received.

### Dose escalation and toxicity

The most common side effects were nausea and/or vomiting, and headache. None of these were dose-limiting (Table 1b). Grade 1 nausea and/or vomiting was observed in three patients. In two of these the event could be attributed partially to morphine administered for tumour pain before commencement of their SR4554 infusion. In one patient, however, vomiting was clearly related to SR4554. Her symptoms resolved upon termination of SR4554 infusion. None of these three patients had to receive granisetron, however.

One patient experienced a grade 1 itch around his neck within 2 h of SR4554 infusion, which was considered possibly drug-related, which resolved within 24 h.

A mild headache (grade 1) was observed in five patients. In all of these patients, the symptom appeared within 13 h of SR4554 infusion, and was mild in all the cases. It resolved within 24 h of infusion and did not recur. Two of these patients were later found to have brain metastasis 3 and 4 months later, respectively, but given the temporal relationship with SR4554 this symptom was considered possibly drug-related.

One patient experienced alteration in taste sensation within minutes of the start of SR4554 infusion. This was of no clinical significance and was almost certainly due to the DMSO component of the infusion.

No other toxicities were seen. During the follow-up period, we did not observe any haematological or biochemical alterations on blood tests or urinalysis.

### Plasma pharmacokinetic studies

Twenty-five patients underwent plasma sampling for pharmacokinetic studies (Table 2). Only one patient was treated at each of dose levels 1800 and 2200 mg m^−2^; therefore there are no mean values for these dose levels. SR4554 showed a high volume of distribution (mean±s.d.: 34.6±14.7 l) and a short terminal half-life (3.7±0.9 h). This distribution, together with the high excretion of parent drug described below, resulted in high plasma clearance (9.8±4.2 l h^−1^). As expected, the peak concentration of SR4554 (*T*_max_) was observed at the end of infusion, that is, at either 30 or 60 min. Peak plasma concentration (*C*_max_) was 110.2±50.9 mg l^−1^, and the area under the concentration–time curve to last measured time point (AUC_last_) was 415.4±235.7 hr*mg l^−1^.

Some variability was observed in the overall pharmacokinetic parameters between dose levels. The mean plasma half-life varied from 3.1 to 3.8 h depending on the dose level, the mean volume of distribution of drug was between 27 and 45 l, and mean plasma clearance was between 7.7 and 12.7 l h^−1^.

Based on the narrow dose range studied and limited data at 1800 and 2200 mg m^−2^, it was difficult to draw conclusions regarding the linearity of the pharmacokinetics in this range, but plasma clearance was not significantly different at 1400 mg when compared with 2600 mg SR4554 (Table 2).

### Urinary excretion studies

Twenty-four-hour urine collection samples were analysed for SR4554 in order to calculate the percentage of unchanged SR4554 excreted in 24 h. The average percentage urinary excretion of SR4554 for all dose levels was 47±17%. There was a significant difference between the percentage of SR4554 excreted after the administration of 1400 and 2600 mg (58.6±4.9% *vs* 37.3±5.9%, unpaired *t*-test with Welch's correction *P*<0.01).

### Relationship between creatinine clearance and SR4554 plasma clearance

To explore the possibility that differences in patients’ renal function could account for some of the variability in plasma clearance or percentage drug excreted, we performed a linear regression analysis of creatinine clearance against drug plasma clearance and percentage drug excreted. This showed a modest correlation (*r*^2^=0.38, *P*=0.002) between creatinine clearance and drug plasma clearance, but a non-significant correlation between creatinine clearance and percentage drug excreted in urine (*r*^2^=0.04, *P*=0.39).

### Magnetic resonance spectroscopic studies

A total of 24 patients underwent 2–3 MRS studies following SR4554 infusion. MRS no. 1 and MRS no. 2 were performed at the mean time points of 1.1±0.2 and 16.0±0.4 h, respectively, post infusion (Supplementary Table 5). Two patients (patients 28 and 29) underwent MRS no. 2 at ∼12 h post infusion to examine the feasibility of performing localised studies and the probability of obtaining a ^19^F signal on these localised studies. Only unlocalised MRS studies were performed at the first two time points, that is, immediately after infusion and at 12 h; for patient 28, however, the first MRS study was performed at 4.9 h post infusion due to development of some medical complications, which prevented her from undergoing the study immediately after infusion. Only a small ^19^F signal was observed on this patient's MRS no. 1 study, with an SNR of 3.6, whereas on MRS no. 2 there was no significant ^19^F signal detected (SNR was 1.0). For patient 29, the SNR on MRS no. 1 performed 0.9 h post infusion was 17.7; this decreased significantly to 1.5 on MRS no. 2 performed at 11.7 h post infusion (Supplementary Table 5).

**Figure 1:**
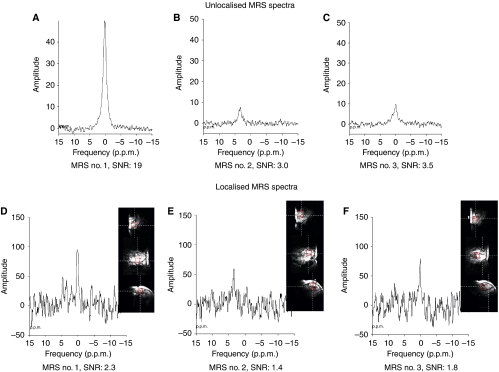
Unlocalised and localised MRS spectra of patient 30. Unlocalised MRS spectra acquired from patient 30 (GIST measuring 62 × 60 × 55 mm) at (**A**) 1.4 h; (**B**) 16.1 h and (**C**) 19.4 h following the end of the SR4554 infusion (2600 mg m^−2^). Localised MRS spectra were acquired at (**D**) 1.8 h; (**E**) 16.4 h and (**F**) 19.7 h following the end of the infusion. In the localised studies, the images in each panel in panels **D**–**F** show different views of the tumour studied, and the highlighted volume (voxel) in each image shows the part of the tumour from where the MRS spectrum was acquired. The timings of ∼16 and ∼20 h post infusion (at the time of MRS no. 2 and MRS no. 3 respectively) are equivalent to approximately 5.5 × and 6.5 × the half-life of SR4554 in this patient, respectively.

Unlocalised MRS no. 3 was performed only for seven patients (whose unlocalised MRS no. 2 showed adequate ^19^F signal to proceed to MRS no. 3), at a mean time point of 19.9±0.7 h post infusion (Supplementary Table 5). A strong ^19^F signal was present in the unlocalised MRS no. 1 studies of all 22 patients as expected, due to the presence of parent SR4554, with a mean SNR of 28.9 (range 3.6–152; excluding patients 28 and 29 who underwent MRS no. 2 at ∼12 h post infusion). At MRS no. 2 a ^19^F signal was present in the unlocalised spectra of 16 of 22 patients, with a mean SNR of 3.1 (range 0.3–10.0), whereas the mean SNR of the detected peaks in unlocalised MRS no. 3 spectra was 3.5 (range 1.8–5.0). Examples of MRS spectra are provided in Figure 1.

Localised MRS studies were performed using spectroscopic imaging for nine patients at the first time point (Table 3). Seven of these patients underwent further localised MRS studies at ∼16 h post infusion (localised MRS no. 2), while two of these patients (patients 28 and 29) had MRS no. 2 at ∼12 h. Five of these nine patients underwent localised MRS at ∼20 h post infusion (localised MRS no. 3). Localised MRS no. 1 was performed at a mean time point of 1.4 h post infusion. A significant SNR (SNR>1.0) was seen for all seven patients, giving a mean SNR of 6.2 (Table 3). Among patients who had localised MRS no. 2 at ∼16 h (performed at a mean time point of 16.4 h post infusion), a significant SNR was seen in five of seven patients, with a mean SNR of 1.7. On localised MRS no. 3, a significant SNR was seen in four of five patients, with a mean SNR of 1.5.

Sixteen patients received a dose of 1400 mg m^−2^. The calculated average tissue concentration of SR4554 from unlocalised measurements at MRS no. 1 in this group was 122±33 *μ*M, as compared with 220±135 *μ*M in plasma.

### Evidence for ^19^F retention

**Figure 2:**
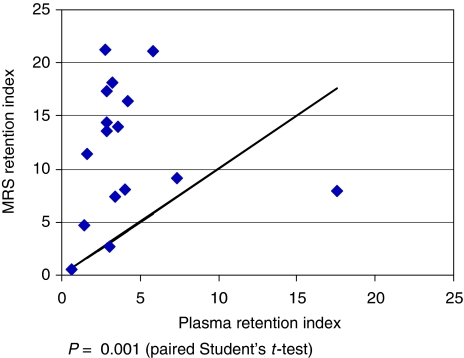
^19^F retention index at 16 h, estimated by MRS, as compared with SR4554 retention index calculated from plasma by HPLC (*n*=16). The diagonal line represents the line of unity (which indicates there is no difference between plasma retention index and MRS retention index). Only one ^19^F-RI (0.45) value lies to the right of the line of unity; this is patient 29 who had MRS no. 2 at 11.7 h, and, therefore, was not expected to show a significant ^19^F-RI at this early time point as this is equivalent to only 3.2 × *T*_1/2_ of SR4554 when the concentration of SR4554 in plasma would exceed its concentration in tumour.

Figure 2 and Table 4 show the SR4554 retention index in tissue calculated using ^19^F MRS, and the equivalent parameter measured in plasma samples acquired before and after each MRS measurement. Comparison of the 16-h retention indices based on MRS-estimated concentrations of SR4554 (mean 13.6, range 0.6–43.7) and plasma pharmacokinetic measurements of SR4554 (mean 4.1, range 0.6–7.3) showed significant ^19^F retention in tumour as detected by MRS (*P*=0.001, paired Student's *t*-test, one-sided; Table 4). This discrepancy between MRS-derived ^19^F-RI and plasma-derived retention index could be attributed to the presence of bioreduced SR4554 in tumour indicating possible presence of hypoxia.

Seven patients underwent unlocalised MRS no. 3 at (mean±s.d.) 19.9±0.7 h (Supplementary Table 5). The dose levels of SR4554 received were 1400 (*n*=1), 2200 (*n*=1) and 2600 (*n*=5). A mean SNR of 3.5 (range 1.8–5.0) was obtained. At this time point, which is equivalent to approximately six times the plasma elimination half-life of SR4554, <1.6% of parent SR4554 would have remained in the tumour vasculature.

The localised MRS studies of five patients performed at ∼20 h post infusion yielded a mean SNR of 1.5 (range 1.0–1.8; Table 3), which is more specifically indicative of ^19^F retention in tumour.

## Discussion

SR4554 is a 2-nitroimidazole that was rationally designed to function as a biomarker of hypoxia detectable by MRS (Aboagye *et al*, 1995b, 1997a, 1997b, 1998a, 1998b). A pilot study demonstrated that it could be administered by IV infusion without toxicity at a dose of 1400 mg m^−2^, above which nausea and vomiting were potentially dose-limiting, at least without prophylactic antiemetics. Preliminary evidence of fluorine retention in tumour was observed, indicative of bioreduction of SR4554 in the presence of hypoxia (Seddon *et al*, 2003).

We now report results of a further 26 patients. In this part of the study, further patients were treated at 1400 mg m^−2^, which was found to be very well-tolerated. Unlocalised MRS studies at this dose level showed some evidence of ^19^F retention suggestive, based on animal model data, of tumour hypoxia, but ^19^F signals detected were generally small. At higher doses of SR4554 (1800–2600 mg m^−2^) administered with prophylactic antiemetics, we observed a higher rate of ^19^F signal detection on both unlocalised and localised MRS studies. As discussed above, any ^19^F signal detected at the time of MRS no. 2 can reasonably be attributed to the presence of bioreduction products, since this study was performed at a time when the concentration of parent compound in plasma would have been only around 3% of *C*_max_ (5 × plasma elimination *T*_1/2_). Another way of looking at this is the SNR. A mean SNR of 3.5 (Supplementary Table 5) is significant for presence of bioreduction products.

Comparison of the 16-h ^19^F-RI provided by MRS with the retention index from plasma pharmacokinetic studies also showed clear evidence of ^19^F retention, particularly in patients for whom localised MRS studies were performed. The mean ^19^F-RI was 13.6, as compared with a mean plasma retention index of only 4.1 (Table 4). The mean ratio of MRS-derived to plasma-derived retention index was 4.8 (range 0.9–18.8) and the ratio was greater than 1 in all but 2 of 16 patients (Table 4). Furthermore, the demonstration of ^19^F signal, albeit of relatively small magnitude, in the 20-h localised MRS study of 4 of 5 patients (Table 3) strongly suggests ^19^F retention, since at this time point an even larger proportion (>98.4%) of parent drug should have been ‘washed out’ and thus any ^19^F signal detected would be likely to originate from bioreduced and bound products of SR4554 in hypoxic cells.

Pharmacokinetic analyses of SR4554 in this study showed results consistent with previously reported data (Seddon *et al*, 2003). SR4554 has been shown to have a short plasma half-life of 3.7±0.9 h, a high plasma clearance of 9.8±4.2 l h^−1^ and relatively high urinary excretion, ensuring rapid elimination of unmetabolised parent compound so that only ^19^F signals from bioreduced products in hypoxic cells are detected by MRS studies performed at the appropriate time. Preclinical studies of SR4554 demonstrated a clear relationship between tumour concentration (AUC) and plasma AUC. Plasma AUC and *C*_max_ increased from 1400 to 2600 mg m^−2^ in this study, as expected from the greater ^19^F signal detected at higher doses. For example, of the seven patients who underwent MRS no. 3, six received higher doses of SR4554, 2200 (*n*=1) and 2600 mg m^−2^ (*n*=5), indicating greater ^19^F retention. Clearly the dose of SR4554 needs to be enough to ensure penetration into tumour to allow sufficient intracellular bioreduction and binding to permit ^19^F quantitation by MRS (Brown and Workman, 1980; Workman and Brown, 1981; Aboagye *et al*, 1996). The modest correlation of creatinine clearance with plasma clearance is consistent with renal excretion of SR4554 by glomerular filtration.

Central nervous toxicities such as disorientation and confusion commonly observed with other nitroimidazoles were not seen in our study, although a very small number of patients experienced grade 1 nausea and vomiting. However, grade 1 headache was observed in five patients shortly after treatment with higher doses of SR4554 (⩾1800 mg m^−2^), which was probably drug-related. Central nervous system penetration was not predicted by preclinical data (Aboagye *et al*, 1995b), which indicated that SR4554 behaves as a hydrophilic agent despite its relatively high octanol : water partition coefficient of 0.65, which was attributed to the hydrophilic character of the side chain (Aboagye *et al*, 1996). Similarly, the pharmacokinetic data are consistent with a hydrophilic drug. Apart from the low incidence of mild nausea/vomiting and headache, SR4554 was found to be well-tolerated even when administered at high doses with prophylactic antiemetics, and hence from these aspects is suitable for use as a diagnostic agent.

In this study, we have not performed correlative studies to confirm the presence of hypoxia as detected by ^19^F MRS. However, the next step would be to correlate results obtained from ^19^F MRS with another method of assessing tumour hypoxia, such as the Eppendorf oxygen electrode or immunohistochemical methods using another nitroimidazole such as pimonidazole. Other hypoxia markers that may be useful for correlative studies with SR4554 include ‘endogenous’ markers of hypoxia, such as HIF-1*α*, CA-9 and GLUT-1, which are upregulated under hypoxic conditions and which can be detected by immunohistochemistry (Vordermark and Brown, 2003). The ultimate evidence for the usefulness of ^19^F MRS as a measure of hypoxia would be to demonstrate its role in predicting patient or treatment outcome, as has been demonstrated with oxygen electrodes in several clinical studies (Brizel *et al*, 1996, 1999; Hockel *et al*, 1996; Nordsmark *et al*, 1996, 2001; Fyles *et al*, 1998; Stadler *et al*, 1999; Rudat *et al*, 2000).

On a case-by-case basis, it is possible that the non-observability of a ^19^F signal in tumour could be due to other tumour-specific factors rather than lack of hypoxia. For example, there are factors at the cellular and biochemical level, such as concentration of the parent nitroimidazole (Chapman *et al*, 1983), temperature (Stratford and Adams, 1977) and the type of nitroreductase enzymes involved (Workman, 1992; Joseph *et al*, 1994), which contribute to the process of nitroimidazole bioreduction and thus generation of a ^19^F signal on MRS. These factors, which may differ significantly in different tumours and individuals, could alter the binding of nitroimidazoles.

In this study, a ^19^F signal was observed at 16 h in patients with the following tumour types: GIST (*n*=9), melanoma (*n*=4), sarcoma (*n*=2), fibromatosis (*n*=1) and head and neck SCC (*n*=1) (Supplementary Table 5). On the other hand, there were three patients with head and neck SCC whose MRS no. 2 did not show a ^19^F signal, in contrast to what one might have expected from the known hypoxic tendency of such tumours (Supplementary Table 5). It is difficult to draw conclusions regarding the correlation between the presence of a ^19^F signal and the histology of the tumour as there are several other factors, which would have contributed to the observation of a ^19^F signal, such as the size and depth of the tumour.

Despite the feasibility and safety of SR4554 as a hypoxia marker as demonstrated in this study, the magnitude and rate of ^19^F detection on localised MRS studies was relatively low despite using higher doses of SR4554. This reflects the inherently low sensitivity of MRS as compared with positron-emission tomography (PET), for example. There is little scope to enhance the sensitivity of ^19^F detection using MRS by further escalating the dose of SR4554 due to solubility limitations. The maximum permitted concentration when diluted in 0.9% saline is 5.4 mg ml^−1^. Doses in excess of 2600 mg m^−2^ would need to be administered in volumes >1 l, which would require infusion times of longer than the 1-h duration used in this study, and which would be inconvenient and potentially harmful in terms of acute fluid load. Other strategies to improve the sensitivity of detection include use of a higher magnetic field strength, for example, 3.0 T, or to increase the number of ^19^F atoms in the SR4554 molecule. To develop SR4554 further as a hypoxia marker, the most practical next step would be to use a more powerful MR scanner.

Positron-emission tomographic imaging has been investigated as a technique for detecting hypoxia. Several clinical studies have been performed to date, which demonstrated the usefulness of pretreatment ^18^F-fluoromisonidazole in predicting patient prognosis or treatment outcome (Eschmann *et al*, 2005; Rajendran *et al*, 2006; Rischin *et al*, 2006). Thus, another possible future development of SR4554 as a hypoxia marker, would be to label the molecule with ^18^F, to enable detection by both ^19^F MRS and ^18^F PET, thus enabling a comparison of retention and hypoxia estimation between the two techniques. This would require a combined PET–MRI scanner, allowing simultaneous acquisition of data from both modalities. The advantage of this is that the intrinsic differences between the two techniques could serve to complement each other in, for example, the sensitivity of detection and spatial resolution. Recent studies have already shown the feasibility of simultaneous PET–MRI imaging in small-animal studies and in humans, although none of these involved hypoxia imaging (Catana *et al*, 2008; Schlemmer *et al*, 2008). Thus, a possible step would be to correlate MRS measurements with ^18^F PET measurements, particularly given recent data on the predictive value of ^18^F PET (Eschmann *et al*, 2005; Rajendran *et al*, 2006; Rischin *et al*, 2006).

There have been recent reports of alternative fluorine-containing nitroimidazoles designed for MRS hypoxia detection (Papadopoulou *et al*, 2006; Procissi *et al*, 2007). One of these is 2-nitro-*α*[(2,2,2-trifluoroethoxy)methyl]-imidazole-1-ethanol, (TF-MISO), an analogue of misonidazole, with chemical properties consistent with a hypoxia marker, which has been shown in preclinical studies to show promise as an MR hypoxia marker (Procissi *et al*, 2007). It would be very interesting to study this compound using MRS in human tumours.

In summary, the results from this study have confirmed the proof of principle for tumour retention of SR4554 using ^19^F MRS and demonstrated the feasibility of this approach that has potential as a non-invasive method of detecting tumour hypoxia. Retention of the agent in many tumours is consistent with hypoxia-mediated bioreduction. The next step would be to correlate results from ^19^F MRS with those from an alternative and accepted method of measuring tumour hypoxia. Further refinements could involve the use of a higher magnetic field strength to increase the sensitivity of detection, or to increase the number of ^19^F atoms in SR4554, or use of simultaneous PET–MRI imaging with a suitably labelled probe molecule.

## Figures and Tables

**Table 1a:** Patient characteristics

	**(*n*=26)**	
**Characteristics**	***n***	**%**
*Age (years)*		
Median	55	
Range	21–83	
		
*Sex*		
Male	16	61.5
Female	10	38.5
		
*WHO performance status*		
0	4	15.4
1	22	84.6
		
*Tumour type*		
Gastrointestinal stromal		
Tumour (GIST)	9	34.6
Head and neck (SCC)	5	19.2
Melanoma	5	19.2
Sarcoma	2	7.7
Fibromatosis	2	7.7
Gastric (adenocarcinoma)	1	3.8
Oesophageal (adenocarcinoma)	1	3.8
Biliary tract (adenocarcinoma)	1	3.8

Abbreviations: GIST=gastrointestinal stromal tumour; SCC=squamous cell carcinoma.

**Table 1b:** Drug-related toxicities: headache (*n*=5), nausea/vomiting (*n*=3), itch (*n*=1) and altered taste (*n*=1)

**Patient**	**SR4554 dose level (mg m^−2^)**	**Toxicity**	**Grade**
12	1400	Itch	1
16	1400	Vomiting	1
19	1400	Nausea	1
25	1800	Headache	1
26	2200	Headache	1
27	2600	Headache	1
28	2600	Nausea, vomiting	1
29	2600	Headache	1
30	2600	Alteration in taste sensation	1
33	2600	Headache	1

**Table 2:** Mean values (±s.d.) of plasma pharmacokinetic parameters and % excreted in urine at various SR4554 dose levels

**Dose level (mg** **m^−2^)**	**No. of patients**	***T*_max_ (h)**	***C*_max_ (mg l^−1^)**	AUC_last_ (h*mg l^−1^)	***T*_1/2_ (h)**	***V*_ss_ (l)**	**Cl (l h^−1^)**	**% excreted**
1400	15	0.9±0.3	98±49^a^	310±160^a^	3.8±0.9	37±17	10.5±4.7^b^	58.6±4.9
1800	1	1.0	74	308	3.1	45	12.7	43
2200	1	1.2	85	291	3.2	38	12.7	35
2600	8	1.0±0.1	141±49^a^	641±231^a^	3.7±1.0	27±6	7.7±2.7^b^	37.3±5.9

Abbreviation: AUC=area under the curve.

a Comparison of patients treated at dose levels 1400 and 2600 mg m^−2^ showed significant correlation both between dose level and AUC (*P*=0.0006), and between dose level and *C*_max_ (*P*=0.05; unpaired Student's *t*-test, two-sided). Patients treated at the intermediate dose levels of 1800 and 2200 mg m^−2^ were not included in this analysis as only one patient was treated at each of these dose levels.

b No statistically significant difference in plasma clearance was observed between the 1400 and 2600 mg m^−2^ groups of patients (*P*=0.14; unpaired Student's *t*-test, two-sided).

**Table 3:** Localised MRS studies, times post-infusion and corresponding SNRs for patients who underwent localised studies at ⩾2 scanning time points (*n*=9)

	**MRS no. 1**		**MRS no. 2**		**MRS no. 3**	
**Patient**	**Time of study post infusion (h)**	**SNR**	**Time of study post infusion (h)**	**SNR**	**Time of study post infusion (h)**	**SNR**
23	1.2	3.2	16.0	1.0	—	—
26	1.0	9.2	16.0	1.0	—	—
28	5.1	2.3	12.2	0.8	—	—
29	1.1	4.9	12.1	1.0	—	—
30	1.8	2.3	16.4	**1.4**	19.7	**1.8**
31	1.7	12.8	16.6	**2.7**	20.0	**1.7**
32	1.2	7.2	16.5	**1.6**	20.7	**1.5**
33	1.4	3.5	16.5	**1.4**	20.8	**1.4**
34	1.3	5.0	16.9	**2.8**	21.3	1.0
Mean^a^	1.4	6.2	16.4	1.7	20.5	1.5
Range	1.0–1.8	2.3–12.8	16.0–16.9	1.0–2.8	19.7–21.3	1.0–1.8
No. of patients with SNR>1.0		9		5		4

Abbreviations: MRS=magnetic resonance spectroscopy; SNR=signal-to-noise ratio.

SNR values >1.0 on MRS no. 2 or MRS no. 3 (highlighted) are considered significant and indicative of the presence of hypoxia.

a Excluding patients 28 and 29 who had MRS no. 2 at ∼12 h post infusion.

**Table 4:** MRS-derived and plasma-derived retention indices at 16 h

**Patient**	**Plasma-derived retention index (RI) at 16 h^a^**	**MRS-derived retention index (RI) at 16 h^b^**	**Ratio of MRS-derived RI to plasma-derived RI**
9	2.8	14.4	5.1
11	7.3	9.1	1.3
12	2.9	13.5	4.7
14	3.6	14.0	3.9
15	5.8	21.1	3.6
16	0.6	0.6	0.9
18	1.5	4.7	3.2
20	3.1	2.6	0.9
23	1.6	11.5	7.2
25	2.9	17.3	6.1
26	3.3	18.1	5.6
30	2.8	21.3	7.6
31	4.1	8.1	2.0
32	4.2	16.4	3.9
33	2.3	43.7	18.8
34	3.4	7.4	2.2
Mean	4.1	13.6	4.8
Range	0.6–7.3	0.6–43.7	0.9–18.8

Abbreviation: MRS=magnetic resonance spectroscopy.

MRS-derived ^19^F-RI at 16 h plotted against plasma-derived retention index for patients for whom there are complete data sets for these time points (*n*=16).

a Plasma-derived retention index=Ratio of plasma SR4554 concentration at the time of MRS no. 2 to its concentration at the time of MRS no. 1 (plasma SR4554 concentration measured by HPLC).

b MRS-derived ^19^F-RI=Ratio of SR4554 concentration estimated by MRS no. 2 to its concentration estimated by MRS no. 1.

## Supplementary Material

Supplementary Table 5

Supplementary Table Caption
