# Supplementary material for: A phase I study of the nitroimidazole hypoxia marker SR4554 using 19F magnetic resonance spectroscopy
Source: Br J Cancer. 2009 Nov 24;101(11):1860–8. doi: 10.1038/sj.bjc.6605425 (PMC2788261; doi:10.1038/sj.bjc.6605425)
Supplement: Supplementary Table 5 [file 6605425x1.doc]

**Supplementary Table 5**

| **Patient** | **SR4554 dose level**  **(mg/m^2^)** | **Tumour type** | **MRS #1** | | **MRS #2** | | **MRS #3** | |
| --- | --- | --- | --- | --- | --- | --- | --- | --- |
|  |  |  | **Time of study post-infusion (h)** | **SNR** | **Time of study post-infusion (h)** | **SNR** | **Time of study post-infusion (h)** | **SNR** |
| 9 | 1400 | Melanoma | 0.8 | 14.6 | 15.8 | 2.1 | - | - |
| 10 | 1400 | Tonsil SCC | - | - | - | - | - | - |
| 11 | 1400 | Liposarcoma | 1.2 | 51.3 | 16.8 | 2.0 | **-** | **-** |
| 12 | 1400 | Dermatofibrosarcoma | 0.9 | 18.5 | 15.4 | 3.3 | **-** | **-** |
| 13 | 1400 | Adenocarcinoma of the ampulla | 1.2 | 6.2 | 16.0 | 0.3 | **-** | **-** |
| 14 | 1400 | GIST | 1.1 | 48.0 | 15.6 | 10.0 | **-** | **-** |
| 15 | 1400 | GIST | 1.0 | 12.5 | 15.8 | 3.2 | **-** | **-** |
| 16 | 1400 | GIST | 1.0 | 152.0 | 15.7 | 2.0 | **-** | **-** |
| 17 | 1400 | Melanoma | 1.3 | 14.0 | 17.0 | 0.5 | **-** | **-** |
| 18 | 1400 | Fibromatosis | 1.3 | 11.7 | 16.4 | 1.3 | **-** | **-** |
| 19 | 1400 | Oesophageal adenocarcinoma | - | - | - | **-** | **-** | **-** |
| 20 | 1400 | Fibromatosis | 1.0 | 13.0 | 15.5 | 0.8 | **-** | **-** |
| 21 | 1400 | Tonsil SCC | 1.0 | 16.3 | 16.0 | 1.0 | **-** | **-** |
| 22 | 1400 | Tongue SCC | 0.8 | 7.0 | 16.0 | 0.3 | **-** | **-** |
| 23 | 1400 | Melanoma | 0.9 | 15.3 | 15.8 | 1.6 | 19.0 | 1.8 |
| 24 | 1400 | Head & neck SCC | 0.8 | 11.0 | 15.5 | 0.3 | - | **-** |
| 25 | 1800 | GIST | 0.9 | 10.4 | 15.6 | 2.7 | - | **-** |
| 26 | 2200 | Melanoma | 0.9 | 56.0 | 15.7 | 8.0 | 19.5 | 4.0 |
| 27 | 2600 | Melanoma | 1.2 | 37.3 | 15.7 | 3.0 | - | **-** |
| 28 | 2600 | Gastric adenocarcinoma | 4.9 | 3.6 | 11.8 | 1.0 | - | **-** |
| 29 | 2600 | Head & neck SCC | 0.9 | 17.7 | 11.7 | 1.5 | - | **-** |
| 30 | 2600 | GIST | 1.4 | 19.0 | 16.1 | 3.0 | 19.4 | 3.5 |
| 31 | 2600 | GIST | 1.4 | 23.0 | 16.3 | 2.5 | 19.8 | 2.5 |
| 32 | 2600 | GIST | 0.9 | 43.0 | 16.1 | 9.0 | 20.3 | 5.0 |
| 33 | 2600 | GIST | 1.2 | 19.0 | 16.1 | 4.5 | 20.5 | 3.5 |
| 34 | 2600 | GIST | 1.0 | 37.0 | 16.6 | 7.0 | 20.9 | 4.5 |
| Mean |  |  | 1.1* | 28.9* | 16.0* | 3.1* | 19.9 | 3.5 |
| SD |  |  | 0.2* |  | 0.4* |  | 0.7 |  |
| Range |  |  |  | 3.6-152* |  | 0.3-10* |  | 1.8-5.0 |
| No of patients who had MRS | | | | 24 |  | 24 |  | 7 |
| No of patients with SNR >1.0 | | | | 24 |  | 17 |  | 7 |
