# Supplementary material for: A phase I study of the nitroimidazole hypoxia marker SR4554 using 19F magnetic resonance spectroscopy
Source: Br J Cancer. 2009 Nov 24;101(11):1860–8. doi: 10.1038/sj.bjc.6605425 (PMC2788261; doi:10.1038/sj.bjc.6605425)
Supplement: Supplementary Table Caption [file 6605425x2.doc]

Supplementary Table 5

**Summary of the timing of unlocalised MRS studies and corresponding signal-to-noise ratios (SNR; n=24). SNR >1.0 on MRS #2 and MRS #3 (highlighted) are considered significant.**

A total of 26 patients received SR4554 infusion, and 24 patients underwent MRS studies (two patients did not have MRS due to technical problems)

- denotes MRS study not performed.

* Excluding Patients 28 & 29 whose MRS #2 was performed at ~12 h post-infusion.
